# Internal Jugular Venous Thrombosis With Superior Vena Cava Syndrome: A Rare First Presentation of Gray Zone Lymphoma

**DOI:** 10.7759/cureus.37096

**Published:** 2023-04-04

**Authors:** Prabasha Weeraddana, Nisha Nepal, Mohammed Saleh, Fnu Sandeep, Niwanthi Weerasooriya, Mehndi Dandwani, Eric Ma

**Affiliations:** 1 Internal Medicine, Danbury Hospital, Danbury, USA; 2 Pathology, Danbury Hospital, Danbury, USA; 3 Hematology and Oncology, Danbury Hospital, Danbury, USA

**Keywords:** classical hodgkin lymphoma, diffuse large b-cell lymphoma, thrombosis, anticoagulation, hilar lymphadenopathy, mediastinal masses, superior vena cava (svc) syndrome, thrombosis of the internal jugular vein, gray zone lymphoma

## Abstract

Gray zone lymphoma (GZL) is defined as a B-cell lymphoma with intermediate features between both diffuse large B-cell lymphoma (DLBCL) and classical Hodgkin lymphoma (CHL). GZL is an aggressive disease, which in addition to the B-symptoms, can present as shortness of breath and neck swelling from underlying superior vena cava (SVC) syndrome. Thrombosis of the internal jugular vein (IJVT) is rare and usually associated with head and neck infection, intravenous (IV) drug abuse, and central venous catheter placement. GZL’s initial presentation as IJVT with SVC syndrome is very uncommon. We report the case of a 47-year-old female presenting with neck swelling and shortness of breath. Initial investigations were oriented at the thyroid gland. A computerized tomography (CT) scan of the chest, neck, and head showed a large anterior/superior mediastinal soft tissue mass with left IJVT. An excisional biopsy of the left axillary lymph node confirmed the diagnosis of GZL. The mediastinal lymphoma can compress the internal jugular vein and also release thrombogenic substances that can cause IJVT. The compression of the SVC by the lymphoma and the IJVT formation can cause SVC syndrome. Both of these conditions can be life-threatening and should be identified in the early stages to prevent complications.

## Introduction

Gray zone lymphoma (GZL) is a rare form of lymphoma that shares characteristics with both diffuse large B-cell lymphoma (DLBCL) and classical Hodgkin lymphoma (CHL) but cannot be classified as one or the other [1]. GZL is more frequent in males aged 20-40 years [2]. It may or may not have mediastinal involvement, but in a mediastinal GZL, it can cause mechanical compression of the internal jugular vein and also produce thrombogenic substances that can lead to Internal jugular venous thrombosis (IJVT) and this can further lead to superior vena cava (SCV) syndrome [3]. These conditions should be identified early, or they can become medical emergencies [4]. Common complications of IJVT include pulmonary embolism, intracranial hypertension, chylothorax, and septic embolization. GZL presenting first with IJVT and SVC without B symptoms is rare and poses a diagnostic challenge. Here we present a case of a left IJVT occurring as a complication of GZL.

## Case presentation

A 47-year-old Caucasian female without significant past medical history presented to the primary care physician (PCP) complaining of painless neck swelling and unexplained weight loss of about 25 pounds in three months. Her family history was significant for bone cancer in her father. She was a non-smoker and she last saw her PCP two years ago.

An examination at the PCP revealed swelling at the left part of the thyroid gland. Routine blood workups and an ultrasound thyroid scan were done. A blood workup at the PCP detected microcytic hypochromic anemia (hemoglobin of 10.8 g/dL (12.1-15.1 g/dL), mean corpuscular volume of 78 fL (80-95 fL) with a low iron level at 27 mcg/dL (60-170 mcg/dL) and normal thyroid stimulation hormone (TSH) of 1.54 mIU/L (0.5-5.0 mIU/L). She had hemoglobin electrophoresis for further investigation, which came back normal. Her stool occult testing was negative. The thyroid ultrasound scan was without any concerning features. The patient was referred to gastroenterology (GE) for new-onset anemia and weight loss. While she was waiting for a GE appointment, she noticed that her neck swelling had rapidly increased for the past 10 days, now involving her face. Because of this, she went to urgent care and was advised to see an otolaryngologist (ENT). Her neck swelled to the point where she felt that she was being strangled with mild difficulty in breathing. Therefore, she again went to see her PCP, where she got a computerized tomography (CT) scan of the head, neck, and chest with contrast.

**Figure 1:**
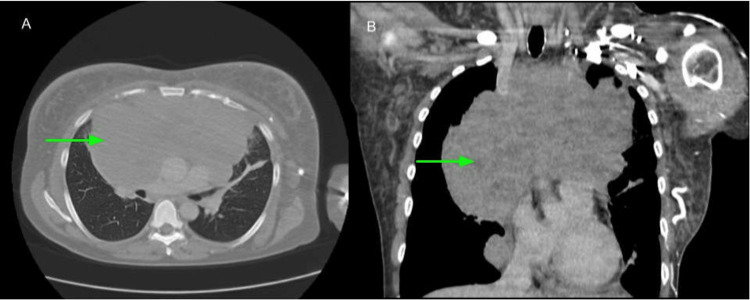
CT scan of the chest with contrast (A: axial view; B: coronal view) showed a large anterior mediastinal mass (green arrows) extending to both hilar regions.

**Figure 2:**
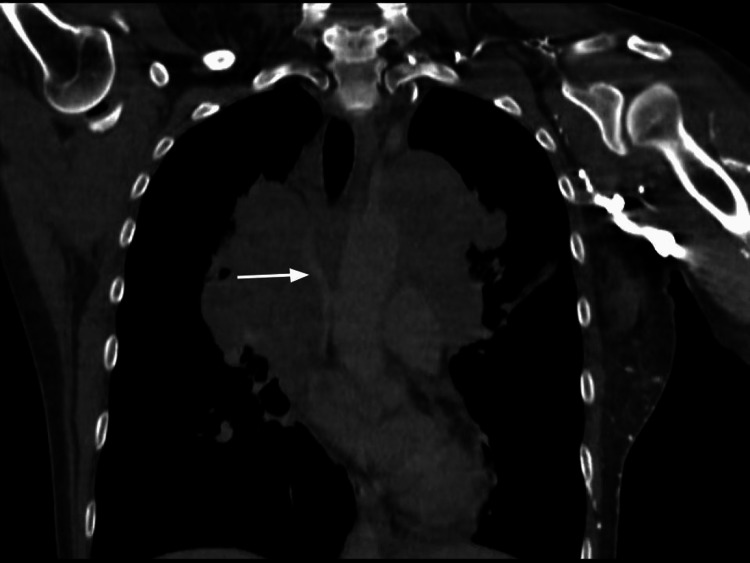
CT scan of the chest with contrast (sagittal view) showed marked narrowing of the mid-to lower-level superior vena cava (white arrow).

**Figure 3:**
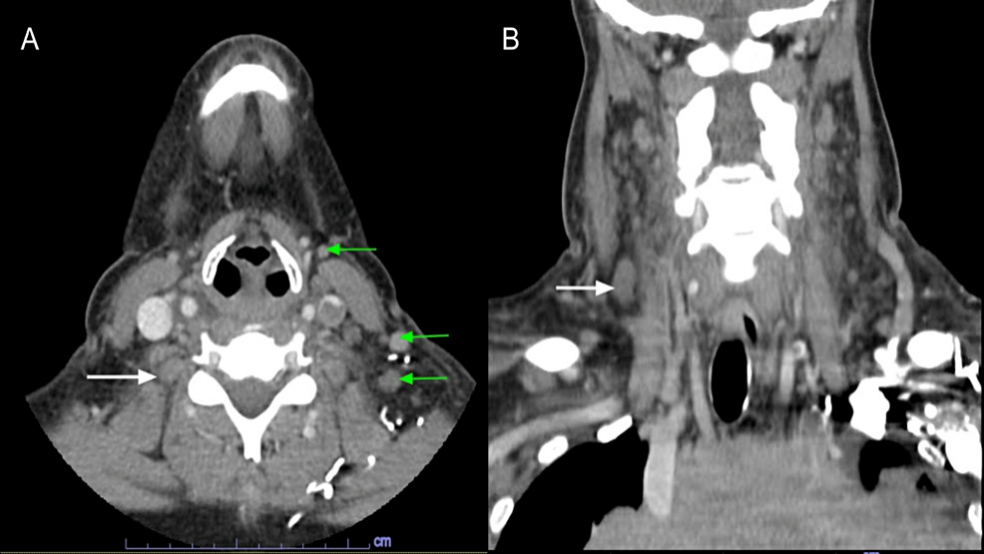
CT head and neck with contrast (A: axial view; B: coronal view) showed bilateral enlarged cervical lymph nodes (green arrows), with the largest within the right internal jugular chain (white arrows).

**Figure 4:**
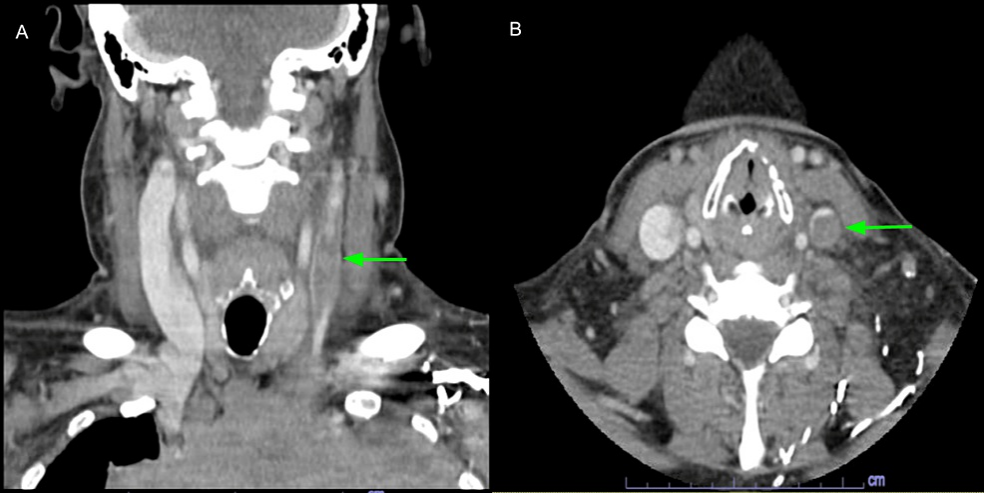
CT head and neck with contrast (A: sagittal view; B: axial view) showed lower left internal jugular vein expansion with internal hypodensity consistent with thrombosis (green arrows).

The CT scan of the chest, neck, and head showed a large anterior/superior mediastinal soft tissue mass likely representing confluent lymphadenopathy. It measured up to 19.2 cm and extended into the perivascular space and both hilar regions (Figure 1). The mass effect caused marked narrowing of the mid-to lower-level SVC and left brachiocephalic vein (Figure 2). The bilateral cervical lymph nodes were scattered and prominent, with the largest within the right internal jugular chain measuring 1 x 1.1 x 1.7 cm (Figure 3), and lower left internal jugular vein expansion with internal hypodensity consistent with thrombosis (Figure 4). Given the CT finding, the patient was referred to the emergency department (ED).

At the ED, her temperature was 36.5°C, her heart rate was 102 beats per minute, her blood pressure was 132/76 mmHg, her respiratory rate was 18 breaths per minute, and her oxygen saturation was 99% on room air. Examination revealed diffuse swelling on the bilateral neck, bilateral cervical lymphadenopathy, and left axillary lymphadenopathy. Her complete blood count showed hemoglobin of 10 g/dL (12.1-15.1 g/dL), a WBC count of 5.2 x 109/L (4.5-11.0 × 109/L), normal renal and liver function tests, and a normal electrocardiogram. Prothrombin time, activated partial thromboplastin time, and the international normalized ratio (INR) were within normal limits.

**Figure 5:**
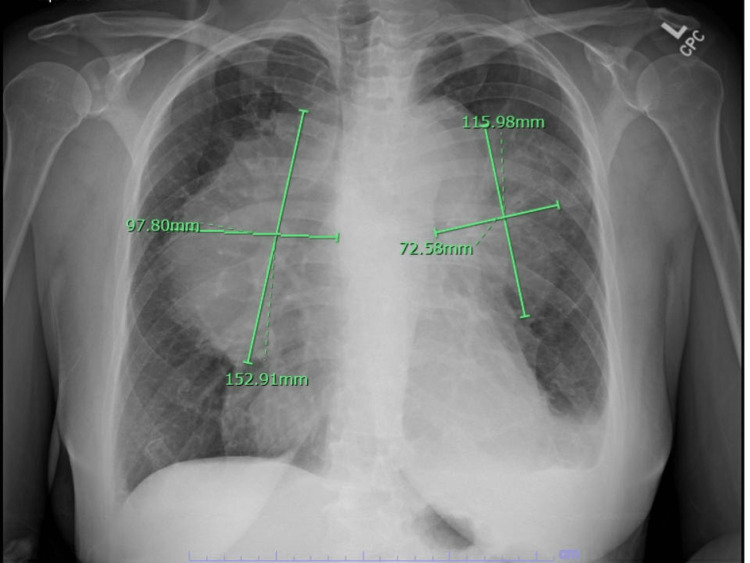
Chest X-ray showed bilateral perihilar masses measuring up to approximately 15.3 x 9.8 cm on the right and 11.6 x 7.2 cm on the left.

The chest X-ray on admission showed advanced bilateral perihilar masses measuring up to approximately 15.3 x 9.8 cm on the right and 11.6 x 7.2 cm on the left (Figure 5). The findings suggested lymphadenopathy. The patient was started on an IV heparin drip. The patient underwent an urgent left axillary lymph node excisional biopsy and was started on dexamethasone 40 mg per oral for four days to reduce the compression on the SVC. 

**Figure 6:**
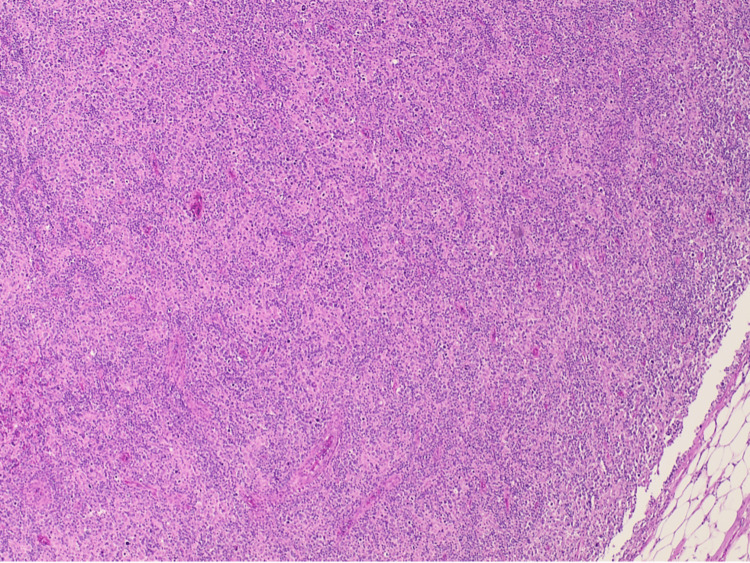
Histology of left axillary lymph node excisional biopsy showed (H&E x 4) obliteration of follicular architecture by the diffuse proliferative process. H&E: hematoxylin and eosin stain

**Figure 7:**
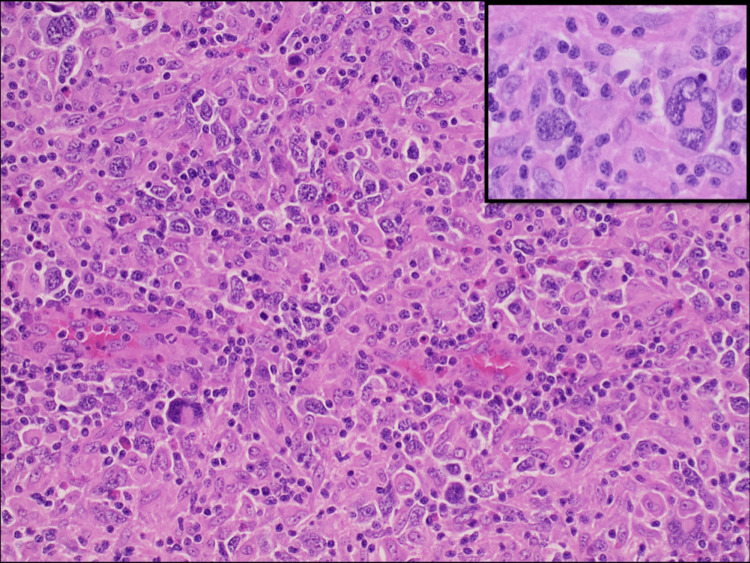
Histology of left axillary lymph node excisional biopsy showed (H&E x 10) diffuse proliferation of very large atypical lymphoid cells with round, irregular, or lobulated nuclei, stippled or vesicular chromatin, variably conspicuous nucleoli, and a moderate amount of pink cytoplasm. Binucleated and multinucleated large, atypical cells were present (left upper corner). Mitotic figures were frequent. H&E: hematoxylin and eosin stain

The patient's pathology report from the left axillary lymph node excisional biopsy showed diffuse proliferation of very large atypical lymphoid cells with round, irregular, or lobated nuclei, stippled or vesicular chromatin, variably conspicuous nucleoli, and a moderate amount of pink cytoplasm. Binucleated and multinucleated large, atypical cells were present. Mitotic figures were frequent. The large atypical cells appeared to involve the subcapsular sinus.The large atypical cells were CD45-, CD20-, CD19-, CD79a-, PAX5+ (weak), CD30+ (diffuse, strong), CD15+, CD10-, BCL6-, MUM1+, CD21 + (weak), EBV immunostain-, CD138-, ALK1-, granzyme-, EMA-, p53+ (diffuse, strong), CD23-, with most large atypical cells positive for Ki-67. Lymphoid cells were negative for cyclin D1. The majority of small lymphocytes were CD3+, CD5+, and CD7+ T cells with a mixture of CD4+ and CD8+ T cells. Also, there were scattered and clustered CD20+ small B cells within the infiltrate. BCL2 stains many small lymphocytes and weakly stains some of the large atypical cells (Figures 6, 7). Her immunophenotypic findings were typical for CHL. But in contrast, the histologic findings were not typical of Hodgkin lymphoma. The neoplastic cells did not closely resemble Reed-Sternberg cells and variants. They were atypical lymphoid cells that were much larger, more numerous, and more pleomorphic than seen in the usual DLBCL. Sinusoidal involvement was distinctly uncommon for Hodgkin lymphoma. Thus, there was a disconnect between the histologic features and the immunophenotype. Therefore the lymphoma, associated with a very large symptomatic mediastinal mass, was best classified as mediastinal GZL.

A bone marrow biopsy was obtained and excluded lymphoma involvement. A flow cytometry study revealed that the bone marrow was neither morphologically nor immunophenotypically involved in lymphoma. She was eventually transitioned to Lovenox from a heparin drip while in the hospital. The patient started chemotherapy with the EPOCH (etoposide, prednisone, Oncovin, cyclophosphamide, and doxorubicin hydrochloride) regimen. By the time of the patient’s discharge, her face and neck swelling had significantly improved, and she tolerated the treatment well without experiencing significant toxicity. She transitioned to Xarelto 20 mg daily upon discharge, and she was to closely follow up with an oncologist as an outpatient for the continuation of chemotherapy.

## Discussion

GZL is a rare form of lymphoma and malignancy is a rare course for IJVT. Here we report a case of left IJVT with SVC syndrome as a complication of mediastinal GZL.

GZL shares characteristics with both DLBCL and CHL but cannot be classified as one or the other [1]. GZL is more frequent in males aged 20-40 years [2]. In a study, patients without the involvement of mediastinum at diagnosis were older than those with mediastinal tumors [5]. As GZL shares immunohistochemical traits with both CHL and DLBCL in the same tumor tissue sample, for accurate diagnosis, thorough sampling should be done [2]. Sarkozy et al. noted 139 cases of GZL, of which 86 cases had a morphology more resembling CHL, but with large B cell lymphoma (LBCL) immunohistochemistry (CD20 and/or CD79a, OCT2, BOB1, PAX5) on all tumor cells (CHL-like GZL), while 53 cases had a morphology more resembling LBCL but harbored a CHL immunophenotype (LBCL-like GZL) (5). Patients with GZL may present with B-symptoms (night sweats, weight loss, and fever), swollen lymph nodes, fatigue, loss of appetite, itchy skin, and bleeding. Patients with mediastinal involvement experience pain or pressure in their chests, shortness of breath, hoarseness, and difficulty breathing [6]. In our case, the patient first presented with IJVT and SVC syndrome.

IJVT may be the result of internal jugular vein catheters. The other known reasons for such an occurrence are otolaryngological infections, soft tissue infections around the neck, intravenous drug abuse, recent head/neck surgery, hypercoagulable states such as protein C or S deficiency, factor V Leiden, deficiency of antithrombin III, polycythemia, and hyperhomocysteinemia. Malignant neck masses may also precipitate IJVT(3). There are reported cases of IJVT secondary to a left upper lobe non-small cell lung cancer [7], a soft tissue infection in the vicinity of the internal jugular vein [8], and a deep neck lipoma [9]. There are three causes listed in Virchow's law that lead to thrombus formation-damage to blood vessels, a hypercoagulable state, and irregular blood flow. Both mechanical blood vessel compression caused by tumors and the production of thrombogenic substances can result in thrombus formation [3]. These two are the most likely causes for left IJVT in our patient.

The preferred course of treatment for people with IJVT is anticoagulation. After an IJVT diagnosis, anticoagulant medication can prevent serious side effects, including pulmonary embolism. Anticoagulants include unfractionated heparin, subcutaneous fondaparinux, oral factor Xa inhibitors (rivaroxaban or apixaban), subcutaneous low molecular weight (LMW) heparin, and subcutaneous fondaparinux (UFH). The duration of anticoagulation treatment lasts from four to 12 weeks [10]. Our patient first started on IV heparin and upon discharge switched to Xarelto.

SVC syndrome occurs due to partial or complete obstruction of blood flow through the SVC. The most frequent cause of SVC syndrome is extrinsic compression and blockage of the SVC in the mediastinum. The majority of SVC syndrome cases are caused by mediastinal tumors. And at least 40% of cases of SVC syndrome currently have nonmalignant origins. SVC syndrome is increasingly being caused by pacemaker wires and intravascular catheters. Our patient had a large 19.2 cm confluent lymph nodes mass compressing mid-to lower-level SVC and left brachiocephalic vein. She presented with neck swelling and mild shortness of breath. Her initial workup was directed towards the thyroid gland and then GE causes until her symptoms got severe. At the initial stage of SVC syndrome, clinical features are indistinct. Therefore clinicians should evaluate patients carefully to prevent dreaded complications and identify the causes of SVC syndrome early. SVC syndrome most frequently manifests as face/neck edema, dilated neck veins, cough, dyspnea, swelling of the upper extremities, dilated chest vein collaterals, and conjunctival suffusion. Stridor, hoarseness, dysphagia, pleural effusion, stupor, and coma are other, less frequent signs and symptoms of SVC syndrome [11]. Ultrasound, magnetic resonance imaging (MRI), and radiographic imaging help in the identification, localization, and finding of the cause of obstruction. Although SVC syndrome is a rare occurrence, it can be a serious oncologic emergency. Those who are at high risk for developing this ailment need to be identified by an advanced practitioner. In an ambulatory situation, early detection of imminent SVC syndrome symptoms and indications is crucial [4]. 

We present a case of a 47-year-old non-smoker female who presented with a complaint of neck swelling and unexplained weight loss. A blood workup revealed hypochromic microcytic anemia and a CT neck and chest showed a large anterior/superior mediastinal soft tissue mass, which had compressed SVC and left brachiocephalic vein. Further investigation revealed lymphadenopathy and a biopsy confirmed gray lymphoma. After the biopsy, she was started on dexamethasone to reduce the compression of SCV, intravenous heparin for thrombosis, and chemotherapy with EPOCH regimen for GZL. The patient tolerated the treatment well.

## Conclusions

Here we report the rare complication of rare malignancy, left IJVT with SVC syndrome secondary to GZL. Even though it's an uncommon case diagnosis, clinicians should maintain high index suspicion and do thorough history and clinical examinations as delay in diagnosis can result in serious emergencies. 
